# Circulating miRNAs: A New Opportunity in Bone Fragility

**DOI:** 10.3390/biom10060927

**Published:** 2020-06-18

**Authors:** Simone Donati, Simone Ciuffi, Gaia Palmini, Maria Luisa Brandi

**Affiliations:** 1 Department of Experimental and Clinical Biomedical Sciences “Mario Serio”, University of Study of Florence, Viale Pieraccini 6, 50139 Florence, Italy; simone.donati11@gmail.com (S.D.); simone.ciuffi@unifi.it (S.C.); gaia.palmini@unifi.it (G.P.); 2 Unit of Bone and Mineral Diseases, University Hospital of Florence, Largo Palagi 1, 50139 Florence, Italy

**Keywords:** circulating miRNAs, noninvasive biomarkers, bone fragility, osteoporosis, fracture risk, personalized medicine

## Abstract

Osteoporosis, one of the leading causes of bone fractures, is characterized by low bone mass and structural deterioration of bone tissue, which are associated with a consequent increase in bone fragility and predisposition to fracture. Current screening tools are limited in estimating the proper assessment of fracture risk, highlighting the need to discover novel more suitable biomarkers. Genetic and environmental factors are both implicated in this disease. Increasing evidence suggests that epigenetics and, in particular, miRNAs, may represent a link between these factors and an increase of fracture risk. miRNAs are a class of small noncoding RNAs that negatively regulate gene expression. In the last decade, several miRNAs have been associated with the development of osteoporosis and bone fracture risk, opening up new possibilities in precision medicine. Recently, these molecules have been identified in several biological fluids, and the possible existence of a circulating miRNA (c-miRNA) signature years before the fracture occurrence is suggested. The aim of this review is to provide an overview of the c-miRNAs suggested as promising biomarkers for osteoporosis up until now, which could be helpful for early diagnosis and monitoring of treatment response, as well as fracture risk assessment, in osteoporotic patients.

## 1. Introduction

Bone is a dynamic structure of calcified connective tissue that undergoes constant remodeling. Its main functions are to protect the body structures, offer mechanical support for locomotion, and maintain mineral homeostasis (i.e., calcium, magnesium, and phosphate) [1]. This mineralized tissue is made up roughly of 90% extracellular components, which in turn are comprised of 60% inorganic components (calcium hydroxyapatite crystals), 30% organic components (collagen molecules and noncollagenous proteins), and about 10% blood vessels and cells [2]. The two-sided nature of the bone ensures on one hand to absorb stress through elastic deformation (collagen fibers), and on the other hand to withstand mechanical strains (mineral phase) [3,4].

Bone remodeling is a closely orchestrated process in time and space by the basic multicellular unit of bone (BMU), which consists of osteocytes, osteoclasts, osteoblasts, and bone lining cells [5]. Its key phases are described below [6]:

1. Mechanical stress or biochemical stimuli are detected by osteocytes;
2. Activation results in retraction of bone lining cells and digestion of the collagenous membrane by matrix metalloproteinases;
3. Preosteoclasts are recruited and, following activation, become multinucleated osteoclasts mediating the bone resorption;
4. Osteoblasts reach the resorption cavity producing new osteoid, which in turn calcifies.

A normal bone remodeling is essential to maintain healthy bone and, consequently, its functions throughout its lifetime. Indeed, under normal circumstances, there is no change of bone mass since the amounts of resorbed and synthesized bone are comparable [7].

On the contrary, a disrupted equilibrium of bone resorption and formation can result in a loss of bone mass and a structural deterioration that predisposes the onset of microdamages, thereby increasing the risk of fractures. Fragility fractures occur most commonly in the forearm, hip, and vertebrae, and can cause protracted pain, and impair quality of life, increasing patient morbidity and mortality, thus making them one of the main global social, health, and economic problems [8].

**Scheme 1:**
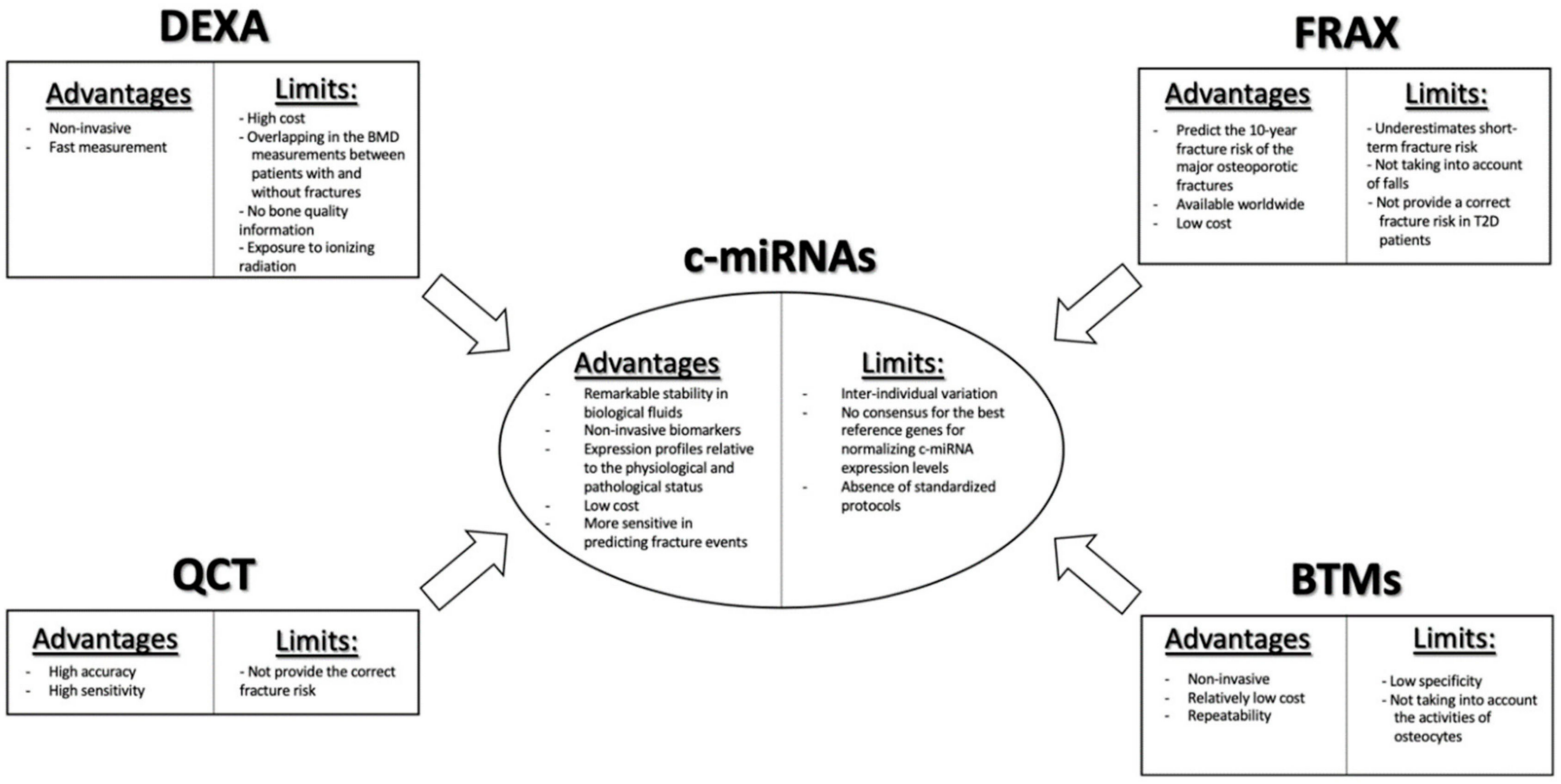
Clinical utility of c-miRNAs as biomarkers for bone fragility and its benefits compared with the classical fragility fracture risk assessment tools.

In clinical routine, the “gold standard” for the prediction of fragility fractures risk is the measurement of bone mineral density (BMD) by using dual-energy X-ray absorptiometry (DEXA) (Scheme 1). Nevertheless, this diagnostic tool has limitations, due to its low detection rate, in discriminating the patients who will suffer fractures, given the great overlap in BMD values among patients with and without fractures. DEXA provides only information regarding the quantity of bone tissue, but not about the changes in bone architecture and mechanical properties. Moreover, it may present a risk of exposure to ionizing radiation [9]. Another useful tool for assessing fracture risk is the Fracture Risk Assessment Tool (FRAX^®^) algorithm, which has been recommended by the World Health Organization (WHO) for estimating the 10-year probability of the major fracture patterns in osteoporotic patients (Scheme 1). The FRAX tool combines different clinical risk factors, including age, gender, body mass index (BMI), smoking, alcohol intake, use of drugs, causes of secondary osteoporosis, prior fragility fractures, family history of fractures, as well as DEXA measurements at the femoral neck, enabling a better prediction of fracture risk than BMD alone [10]. However, even this approach presents some limitations. Indeed, it underestimates short-term fracture risk, does not consider falls, and does not provide a correct fracture risk in type 2 diabetes patients [11,12,13]. Despite the development of novel imaging technologies, such as quantitative computerized tomography (QCT), which can provide information on volumetric BMD, bone geometry, and distinguish between trabecular and cortical bone compartments, redressing the weakness of DEXA, even the latter is not able to capture a relevant portion of fracture risk (Scheme 1) [14].

Nowadays, even biochemical bone turnover markers (BTMs) are being implemented for the assessment of bone formation and resorption (Scheme 1). Currently, the most specific and sensitive BTMs regarding the evaluation of bone formation are bone alkaline phosphatase (ALP), procollagen type I N-terminal propeptide (PINP), and serum total osteocalcin. By contrast, the evaluation of the type 1 collagen metabolites, such as pyridinoline (PYD), deoxypyridinoline (DPD), cathepsin K (CTX, NTX), and matrix-metalloproteases (MMP)-generated (CTX-MMP or ICTP) type I collagen fragments, as well as serum 5b isoenzyme of tartrate-resistant acid phosphatase (TRACP5b), are available for the evaluation of osteoclast bone resorption activity [15,16]. As with measurement of BMD, BTM measurements also present limitations, such as low specificity for bone tissue, given that type I collagen is allocated in different organs, the unsuitability to make a clear distinction between metabolic activity in different skeletal parts, and because they do not take into account the activity of osteocytes, despite the fact that these cells play a key role in the bone remodeling process [16].

In this respect, an increasing effort is required to identify novel biomarkers, which can be used alone or in association with existing biomarkers, to provide a better understanding of bone strength and, consequently, classify patients at high risk of fragility fractures and/or monitor treatment efficacy.

During recent years, small noncoding RNAs have received increasing attention due to their possible use as biomarkers for the assessment of fracture risk and as new treatments for bone fractures, including microRNAs (miRNAs) [17].

In 1993, *lin-4*, which affects development in the *Caenorhabditis elegans* (*C. Elegans*), was the first miRNA discovered by the Ambros and Ruvkun groups [18,19]. In the following years, miRNAs were established in many other organisms, but not in bacteria [20].

These are a class of small noncoding RNAs of approximately 18–25 nucleotides in length, that exert a negative role of gene expression by acting at the post-transcriptional level [21,22]. Currently, about half of all recognized human miRNAs are sited in intragenic regions, mainly within intronic regions, and the remaining are intergenic [20]. At least 20–30% of human transcripts have been estimated to be regulated by miRNAs [23]. In most circumstances, miRNAs interact with the 3′-UTR of mRNAs target, causing their degradation and/or translational repression [24], even if it has been reported that they may bind with other regions, including 5′-UTR, coding sequence, and gene promoters [20]. miRNAs are able to interact with target mRNA, especially via their seed sequence (nucleotides 2–8), inducing either the suppression of protein translation or its degradation, via incomplete or complete complementary, respectively [25,26].

It is possible to distinguish two biogenesis pathways of miRNAs. The canonical biogenesis pathway is the prevalent pathway by which they are transcripted into a long primary miRNA (pri-miRNA) by RNA polymerase II and, subsequently, processed into precursor miRNA (pre-miRNA) by DiGeorge Syndrome Critical Region 8 (DGCR8), an RNA-binding protein, and Drosha, a ribonuclease III enzyme [27]. The resulting pre-miRNAs, identifiable by two nucleotides 3′ overhang, are then exported to the cytoplasm by an exportin 5/RanGTP complex, where they are processed by Dicer, an RNase III endonuclease, to form a small double-strand RNA of about 18–25 nucleotides [27,28]. This duplex is unwound, and the strand with the lowest thermodynamic stability at the 5′ end, termed guide strand, is loaded into the RNA-induced silencing complex (RISC) leading to the establishment of miRNA-induced silencing complex (miRISC) [29,30].

miRISC interacts with its target mRNA through miRNA response elements (MREs) mediating either its degradation or protein translation inhibition, according to the complementarity degree with the target mRNA, as previously mentioned [20].

There is also a noncanonical DROSHA-independent pathway through which their short hairpin RNAs (shRNAs) or miRtrons have an origin. Other pre-miRNAs, such as Ago-2-miRs, complete their maturation by Ago-2 in a Dicer-independent pathway. Finally, DROSHA/Dicer-independent pathways generate molecules, such as endo-siRNA [20].

At present, there are 2654 homo sapiens mature miRNAs registered in the miRBase registry (available online at: http://www.mirbase.org); several of them are involved in various biological processes, such as development, cell differentiation and proliferation, survival, metabolism, and many others [31].

Notoriously, it has been observed that deregulation of their expression profiles is implicated in the onset and progression of various diseases, and that a specific miRNA signature can help in discriminating a normal or pathological tissue condition [32].

Several in vitro and in vivo studies have demonstrated that miRNAs are crucial factors in osteoblastogenesis and osteoclastogenesis processes, and, therefore, deregulation of their expression levels may affect osteoblast, osteoclast, and osteocyte functions with consequent disequilibrium in the bone remodeling. miRNAs are not just involved in regulating bone remodeling, but have also been implicated in fracture repair [17].

In recent years, several studies have focused on a stable circulating miRNA (c-miRNA) form as noninvasive biomarkers (Scheme 1). In 2008, four independent studies demonstrated the existence of stable cell-free mature miRNAs in blood, and showed that the changes in their serum/plasma levels can be indicative of both physiological and pathological states, thus making them useful candidate molecules as noninvasive biomarkers [33,34,35,36]. An aberrant c-miRNA expression profile has been pointed out in several different disease conditions, including cancer, viral infections, cardiovascular and muscular disorders, nervous system disorders, and diabetes [8]. Moreover, increasing evidence suggests that an altered BMD status, as well as the presence of bone fractures, might be reflected in the changes of c-miRNA expression profiles. Fractures can occur due to direct or indirect trauma, repeated microtrauma, and osteoporosis, one of the most important causes of such fractures in the elderly [17].

Additional studies established the appearance of c-miRNAs in a plethora of extracellular biofluids, including cerebrospinal fluid [37], saliva [38], breast milk [39], urine, tears, bronchial lavage, seminal fluid [40], and ovarian follicular fluid [41].

Moreover, to the contrary of cellular RNA species, their remarkable stability in biological fluids has been shown, despite a high RNase activity, and the ability to resist even in harmful conditions (e.g., boiling, multiple freeze–thaw cycles, and high or low pH boiling, multiple freeze–thaw cycles and high or low pH) [35,36]. Their notable stability in extracellular fluids has been hypothesized to be imputable to an association with proteins, such as Argonaute 2 (Ago2), high-density lipoproteins (HDLs), and nucleophosmin 1 (NPM1) [42,43,44], or encapsulation in membrane-bound vesicles, such as shedding microvesicles (1µm), exosomes (50–90 nm), and apoptotic bodies [38,45].

Despite the fact that intercellular communication was limited to the cell-cell communication (i.e., gap junction or signal transduction factors) not so long ago, several studies have indicated the possible involvement of these noncoding RNA molecules as a new way of intercellular communication: vesicle-encapsulated miRNAs may enter cells by phagocytosis, endocytosis, or direct fusion with the plasma membranes, while vesicle-free miRNAs may be carried by specific proteins and taken by specific receptors on the recipient cell surface [42,46,47].

However, to date, there is no one outlook regarding the origin and biological function of c-miRNAs.

The identification of c-miRNA-specific expression profiles would be critical not just for valid diagnostic strategies, but also for therapeutic utility, such as for the treatment of bone loss, and even to accelerate the physiological fracture repair process.

**Figure 1:**
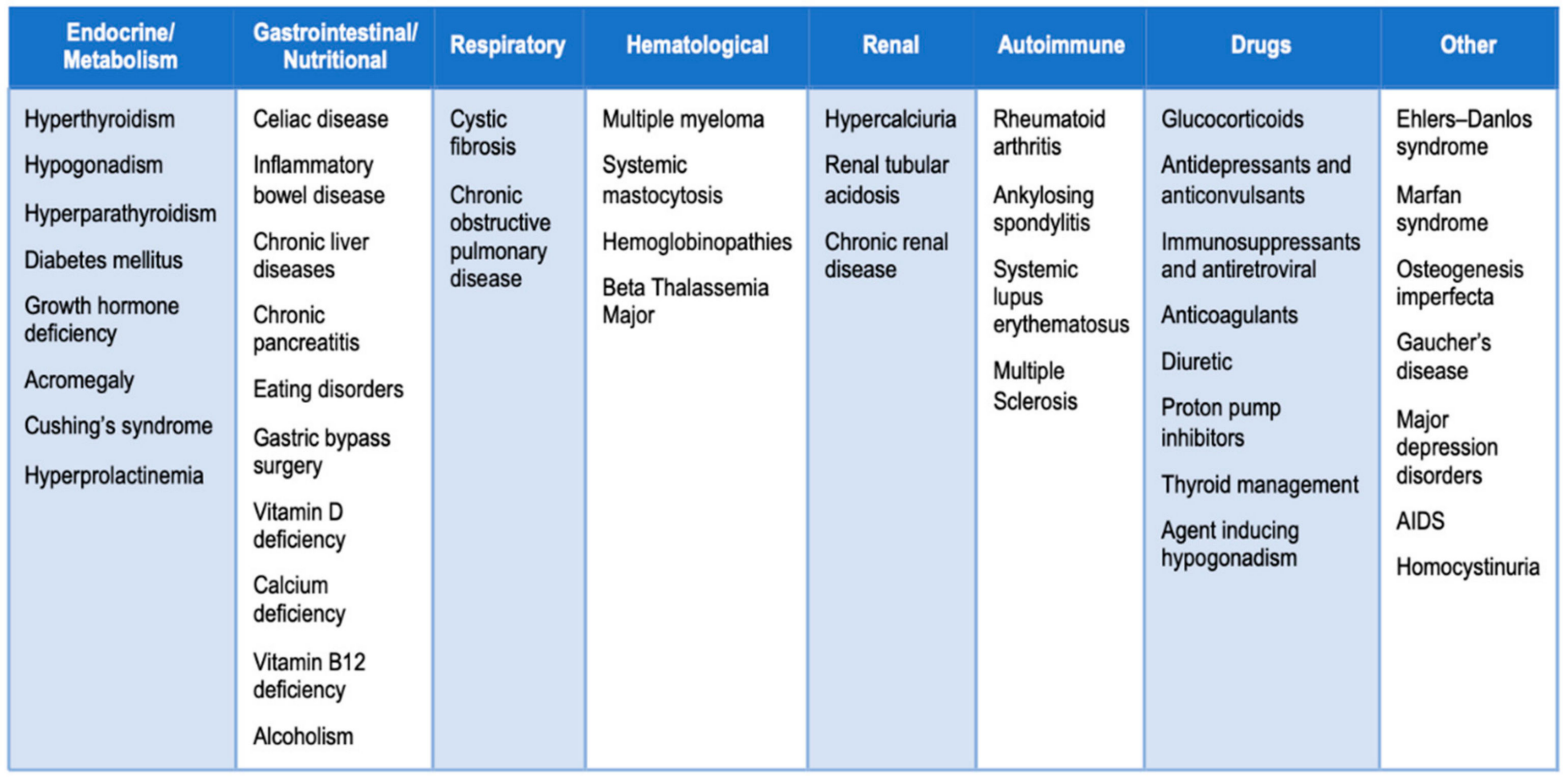
Common causes of secondary osteoporosis. Osteoporosis can be divided into two major subtypes: primary osteoporosis is a condition that develops in postmenopausal women and in elderly people, while secondary osteoporosis is referred as bone loss caused by specific clinical diseases or therapies [12,48,49].

In this context, several studies have evaluated the expression levels profile of c-miRNAs in patients with osteopenia, osteoporosis, or fragility fractures. We carried out a literature search for potential miRNAs as biomarkers in bone fragility by using different combinations of significant keywords: “miRNA”, “Circulating miRNA”, “Bone fragility”, “Osteoporosis”, “different causes of secondary osteoporosis (Figure 1)”, and “monocytes”.

In the first section, we have summarized the studies regarding c-miRNAs, which could be potential biomarkers for diagnostic and/or therapeutically utility for bone fragility to date. Given that bone fragility can result from an excessive resorption activity led by osteoclasts that overlook the bone formation carried out by osteoblasts, in the second section we have taken into account the most remarkable scientific papers relative to the expression analysis of miRNAs isolated from the circulating monocytes of subjects with high BMD, because these cells are osteoclast precursors, and, therefore, are directly involved in the osteoclastogenesis process. Since these cells appear in peripheral blood, they could be noninvasively isolated, and their miRNA profile could be analyzed to identify miRNA biomarkers in monocytes isolated from postmenopausal women with osteoporosis.

## 2. Circulating miRNAs as Potential Biomarkers in Bone Fragility

### 2.1. Osteopenic/Osteoporotic Patients

For the first time, in 2014 Seeliger et al. [50] investigated the potential role of c-miRNAs as biomarkers in osteoporosis. In particular, they sought to identify a serum miRNA signature able to discriminate osteoporotic (OP) patients with hip fractures from those fractured non-OP. First, they screened 83 different miRNAs by using miRNA PCR arrays between two pooled serum samples from 10 OP samples and 10 non-OP samples, respectively. Out of these, the expression levels of 11 miRNAs (miR-21-5p, miR-23a-3p, miR-24-3p, miR-25-3p, miR-27a-3p, miR-100-5p, miR-122a-5p, miR-124-3p, miR-125b-5p, miR-148a-3p, and miR-223-3p) were significantly higher in the serum of OP patients compared with the other group. Therefore, a validation step was performed in an independent cohort of 30 OP with fractures and 30 non-OP patients by using Real-Time Quantitative Reverse Transcription PCR (qPCR). Along with the 11 miRNAs, they also analyzed miR-93 and miR-637, because the latter has been associated with bone development. They identified nine differentially expressed miRNAs (miR-21-5p, miR-23a-3p, miR-24-3p, miR-93, miR-100-5p, miR-122-5p, miR-124-3p, miR-125b-5p, and miR-148a-3p) between samples of OP patients and non-OP patients. Receiver-operating characteristic (ROC) analysis revealed that all nine identified serum miRNAs had significant Area Under the Curve (AUC) values and, in particular, the associated AUC of miR-122a was the highest in distinguishing OP patients from the non-OP group, reaching 0.77. Then, they evaluated the same miRNAs in bone tissue samples of OP patients, observing the upregulation of six miRNAs (miR-21-5p, miR-23a-3p, miR-24-3p, miR-25-3p, miR-100-5p, and miR-125b-5p). In conclusion, the expression levels of five miRNA (miR-21-5p, miR-23a-3p, miR-24-3p, miR-100-5p, and miR-125b-5p) were significantly higher in both serum samples and bone tissue. These results suggested their potential role as novel biomarkers for osteoporosis and their crucial role in the pathogenesis of osteoporosis-associated hip fractures.

Li et al. [51] examined the expression levels of miR-21-5p, miR-133a, and miR-146a, which have been previously reported to be important in osteoporosis, in the plasma of 40 OP patients, 40 osteopenic patients, and 40 healthy controls (HC), by using qPCR. miR-21-5p levels were lower, while miR-133a levels were higher in the plasma of OP and osteopenic patients compared with HC, observing moreover a significant correlation between these two miRNAs and BMD values. On the contrary, miR-146 levels were not differentially statistically expressed among the population of the study. Overall, they found that miR-21-5p and miR-133a could be used as potential diagnostic biomarkers in postmenopausal osteoporosis.

The purpose of a study by Panach et al. [52] was to identify differentially expressed miRNAs in the serum of OP patients with hip fractures compared to osteoarthritic patients, the latter chosen as control group. In the profiling stage, they analyzed 179 miRNAs in two pooled serum samples derived from eight fractured patients at the femoral neck and five osteoarthritis controls by using qPCR. From this analysis, five candidate miRNAs (miR-143-3p, miR-122-5p, miR-125b-5p, miR-210, miR-21-5p) were selected according to the Benjamini–Hochberg false discovery rate (FDR) correction. In addition, they also selected miR-34a-5p since this miRNA has been suggested as an osteoclast key suppressor in the literature. These six miRNAs have been validated by using qPCR in the serum of 15 patients with hip fractures and 12 osteoarthritis controls. The expression levels of miR-122-5p, miR-125b-5p, and miR-21-5p were significantly upregulated in the fractured OP group compared with HC. These three miRNAs have proven in a ROC analysis that they have the highest discriminating power between OP patients with hip fractures and the control group. Overall, their findings revealed miR-122-5p, miR-125b-5p, and miR-21-5p as possible biomarkers to distinguish people who suffered a hip fracture from controls, highlighting in particular miR-21-5p.

The purpose of a study by Weilner et al. [53] was to study whether the changes in the serum miRNA expression levels in recent OP fracture patients could be associated with bone metabolism. In the first phase, they analyzed a set of 175 miRNAs in serum samples of seven patients with recent OP fractures at the femoral site versus seven age-matched HC. Six miRNAs (miR-10a-5p, miR-10b-5p, miR-133b, miR-22-3p, miR-328-3p, and let-7g-5p) were differentially expressed as a result of recent femoral neck fracture and may allow the appropriate categorization as fracture or HC samples. These miRNAs have been subsequently validated in an independent cohort of 12 fractured patients compared to 11 age-matched HC by using qPCR. Out of these six miRNAs, only miR-22-3p, miR-328-3p, and let-7g-5p have proved to be significant in the validation analysis, resulting downregulated in the serum of low-trauma OP patients compared with HC. Finally, the authors tested in vitro these three and other miRNAs, selected according to literature, in human mesenchymal stem cells (hMSCs) to study their involvement in the osteogenic process, showing that five of eight proved miRNAs (let-7g-5p, miR-100-5p, miR-10b-5p, miR-148a-3p, and miR-328-3p) were involved in the osteogenic differentiation.

Due to the multifactorial nature of bone fragility in idiopathic osteoporosis, Kocijan et al. [54] investigated a panel of 187 serum miRNAs in 36 idiopathic low-traumatic fractured patients (10 premenopausal women, 10 postmenopausal women, and 16 men) compared with 39 free-fractures HC, by using qPCR to identify a miRNA signature able to distinguish these two states. Among them, a subset of 19 of 91 regulated miRNAs was significantly deregulated between the fractured and healthy groups. ROC analysis indicated that the combination of eight miRNAs (miR-152-3p, miR-30e-5p, miR-140-5p, miR-324-3p, miR-19b-3p, miR-335-5p, miR-19a-3p, miR-550a-3p) had the best ability to distinguish between patients with low-traumatic fractures from the HC, as shown by the highest AUC value. In conclusion, these molecules could be potential biomarkers for the diagnosis of idiopathic osteoporosis in premenopausal women and male patients, as well as for estimating fragility fracture risk. In particular, the authors emphasized miR-29b-3p because this miRNA was reported as a regulator of osteogenic differentiation.

Feichtinger et al. [55] aimed to evaluate the association between serum levels of 19 previously reported bone-related miRNAs, bone microstructure, and bone histomorphometry in 36 idiopathic OP patients with peripheral or vertebral low-trauma fractures. Three miRNAs (miR-29b-3p, miR-324-3p, and miR-550a-3p) were statistically correlated to histomorphometric and microstructure parameters according to the Spearman analysis (*p* < 0.05). In addition, the authors observed decreased expression levels of miR-29b-3p and miR-324-3p in patients undergoing antiresorptive therapy compared to treatment-free patients, contrary to the expression serum levels of miR-550a-3p. In conclusion, they suggested the above-mentioned miRNAs as potential biomarkers for osteoporosis due to their possible ability to discriminate between patients with and without low-traumatic fractures.

The purpose of Chen et al. [56] was to identify suitable miRNAs as potential noninvasive biomarkers for the diagnosis of osteoporosis in postmenopausal women. To reach this objective, they first performed a microarray analysis on pooled serum samples to identify a reliable reference gene (RG) to normalize expression levels of serum miRNAs in different osteopenic and OP models, including ovariectomized rats (OVX), monkeys and humans, by identifying miR-25-3p as suitable RG. By miRNA microarrays, they also identified 15 differentially expressed miRNAs according to the fold change (≥2) and *p*-value (<0.05). Following qPCR validation, the authors observed that the expression levels of miR-30b-5p were significantly lower in both osteopenic and OP women compared with HC, while miR-103-3p, miR-142-3p, and miR-328-3p were statistically lower only in the OP group. All miRNAs showed a positive correlation with BMD and their AUC values obtained by ROC analysis confirmed their potential as possible noninvasive biomarkers in osteoporosis.

qPCR assay has been employed by Bedene et al. [57] to identify novel potential biomarkers for osteoporosis. They compared the plasma profile of nine miRNAs (let-7d-5p, let-7e-5p, miR-30d-5p, miR-30e-5p, miR-126-3p, miR-148a-3p, miR-199a-3p, miR-423-5p, and miR-574-5p), which were selected from previous data, between 17 OP patients and 57 HC. Among them, miR-148a-3p expression levels were statistically higher in the OP group compared to HC. The results suggested the role of miR-148a-3p as a potential biomarker for this systemic skeletal disease. In addition, they performed a correlation analysis between the expression levels of the nine above-mentioned miRNAs and BMD values, trabecular bone scores, and FRAX. miR-126-3p and miR-423-5 were found to be associated with quality and quantity bone parameters.

Yavropoulou et al. [58] analyzed the serum miRNA expression levels between osteopenic/OP patients (35 with and 35 without vertebral fractures) and 30 HC with normal BMD values and without a history of fractures by using qPCR. Twelve miRNAs were selected according to the literature due to their involvement with bone metabolism. In addition, two miRNAs were selected for the analysis because they were previously reported in circulating monocytes of patients with low BMD. Among them, the expression levels of two miRNAs (miR-124-3p and miR-2861) were significantly higher in the serum of patients compared with HC, while three miRNAs (miR-21-5p, miR-23a-3p, and miR-29a-3p) displayed an opposite trend. Moreover, when they compared the serum miRNA expression levels between osteopenic/OP patients with and without fractures, they found that the expression levels of miR-21-5p were significantly lower in patients who have suffered vertebral fractures compared with those without fractures. The diagnostic value to distinguish between women with and without vertebral fractures was analyzed by ROC analysis. The AUC values were 0.66, 0.63, and 0.61 for miR-21-5p, miR-23a-3p and miR-29a-3p, respectively. In summary, they concluded that the identification of a serum miRNA discriminatory signature in fractured patients with low BMD values represents a crucial step to make better diagnoses and treatment of bone disorders.

Ramirez-Salazar et al. [59] investigated the potential role of serum miRNAs as biomarkers for bone mass reduction, and, therefore, an increase in fracture risk. In the discovery phase, they profiled qPCR arrays to evaluate the expression levels of 754 selected miRNAs in the serum of OP patients and HC. Among them, three miRNAs (miR-23b-3p, miR-140-3p, and miR-885-5p) were selected for the subsequent validation phase in an independent greater population composed of osteopenic (28), OP (26), and OP hip fracture (21) patients, because they showed significant differences between the discovery stage groups, and according to the fold change and *p*-values. In addition, they performed ROC analysis to evaluate the discriminatory ability between OP patients and HC for the aforementioned miRNAs in osteopenia, osteoporosis, and fragility status. Overall, the obtained results suggested that miR-23b-3p and miR-140-3p could be used as candidate biomarkers for assessing the risk of fractures as well as for osteoporosis.

Mandourah et al. [60] investigated the relationship between plasma/serum miRNA expression levels and osteoporosis/low BMD. In the first stage, they profiled miRNA qPCR arrays to evaluate 370 mature miRNAs between three pooled serum samples from four HC, eight osteopenic patients, and nine OP patients, respectively. According to the fold change values (>2), 40 differentially expressed miRNAs from the discovery stage have been validated on 139 serum samples and 134 plasma samples by using qPCR. The results showed that miR-122-5p, in serum samples, and miR-4516, in plasma samples, were significantly differentially expressed miRNAs between the three groups. ROC analysis showed that only miR-4516 had an acceptable significantly diagnostic value for osteoporosis. That analysis also showed that there was a greater diagnostic power when miR-122-5p and miR-4516 were combined. It has also been observed that their expression levels were associated with a positive history of fragility fractures and low BMD in OP patients, showing their potential role as diagnostic biomarkers.

Chen et al. [61] analyzed the expression profile of 150 serum miRNAs between nine OP patients and nine age-matched HC. Based on the results from qPCR assay, six miRNAs (let-7g-5p, miR-133a-5p, miR-328-3p, miR-22-3p, miR-2861, and miR-518d-5p) were downregulated, while five miRNAs (miR-10b-5p, miR-21-5p, miR-125b-5p, miR-23-3p, and miR-100-5p) were upregulated in the serum of OP patients. Human and mouse osteoblast cells were used to test the possible effects on osteoblast differentiation after miRNA transfection. miR-10b-5p was able to produce a significant increase in ALP activity and in the deposition of Ca^2+^ nodules, in contrast to miR-328-3p and let-7g-5p, which inhibited osteoblast differentiation. In addition, miR-328-3p, let-7g-5p, miR-100-5p, and miR-10b-3p were found associated with Wnt signaling, which is critical in osteoblast differentiation and maturation. ROC curve analysis established their diagnostic value to discriminate OP patients from HC. In conclusion, these miRNAs could be possible diagnostic biomarkers and even possible targets for the development of novel drugs for the treatment of osteoporosis.

Wang et al. [62] examined 10 bone metabolism-related miRNAs (miR-7-5p, miR-24-3p, miR-27a-3p, miR-100-5p, miR-125b-5p, miR-128, miR-145-5p, miR-211-5p, miR-144-3p, and miR-122-5p) in both serum and bone samples of 45 fractured OP patients and 15 fractured non-OP patients (control group) by using qPCR. The obtained data showed a significant upregulation of miR-24-3p, miR-27a-3p, miR-100-5p, miR-125b-5p, and miR-122-5p, while the expression levels of miR-144-3p were significantly lower in both serum and bone tissue samples of OP patients compared to control group. miR-128 levels were only significantly upregulated in bone tissue, and miR-145 levels were upregulated only in serum samples. Subsequently, they selected miR-144-3p for further analysis to investigate its effects on osteoporosis pathogenesis. By bioinformatic analysis, they found that *RANK* is a miR-144-3p target gene. Therefore, they evaluated its expression in CD14+ peripheral blood mononuclear cells (CD14+ PBMCs) following transfection with miR-144-3p control, mimic and inhibitor, demonstrating that the overexpression of this miRNA negatively regulates osteoclastogenesis via inhibiting *RANK* gene expression *in vitro*. Overall, this research suggests serum miR-144-3p as a potential diagnostic and therapeutic biomarker for osteoporosis.

Feurer et al. [63] attempted to evaluate the relationship between the expression levels of 32 miRNAs, selected according to the previous literature, and bone turnover, bone density, volumetric BMD, and incident fragility fractures in the serum of 123 premenopausal or postmenopausal women with fragility fractures (1 metatarsal fracture, 40 vertebral fracture, 2 hip fracture, 40 wrist fracture, 18 lower end tibia fracture, 6 proximal humerus fracture, and 1 pelvic fracture), and 559 without fractures, by using qPCR. Out of 32 miRNAs tested, only two miRNAs (miR-145-5p and miR-503-3p) were associated with a positive history of fracture as well as with BTMs, BMD, and microarchitecture parameters, but this association was no longer significant after age-adjustment.

Pickering et al. [64] examined the miRNA expression levels between 217 OP patients with fragility fractures and 217 HC without a history of fragility fractures, in addition to making an assessment of the same serum miRNAs in 183 women with abdominal aortic calcification (AAC). Three miRNAs (miR-26a-5p, miR-34-5p, and miR-223-5p) were selected from predictive programs (i.e., TargetScan, MiRWalk database), according to their relevance in vascular calcification and bone metabolism, and assayed by qPCR. The expression levels of selected miRNAs were not differentially expressed between patients and HC in both cohorts.

The aim of a study by Chen et al. [65] was to analyze the serum miRNA profile between patients belonging to four groups, (13 non-OP/nonsarcopenic patients (NN), 46 OP/Osteopenic patients (OO), 15 sarco-osteopenic patients (SOP), and one sarcopenic patient (SP)), by performing qPCR. Eight bone-related miRNAs (miR-1-3p, miR-21-5p, miR-23a-3p, miR-100-5p, miR-125b-5p, miR-133a-3p, and miR-206) were not differentially expressed among the four groups, nor when compared according to the muscle or bone status (51 sarcopenic patients vs. 51 non-SP, and eight OP patients vs. 55 non-OP), even if fold changes of specific miRNAs indicated upregulation or downregulation in OP patients compared to non-OP subjects. Spearman correlation showed that the relative serum expression levels of miR-125b-5p and miR-23a-3p were statistically positively correlated with age and TRAP5b levels, respectively, while the expression levels of miR-21-5p were significantly negatively correlated with trochanter BMC.

Lin et al. [66] analyzed the expression profile of miR-338 cluster (which includes miR-338-3p and miR-3065-5p), whose deregulation has been observed during in vitro osteoblast differentiation, in the serum of 15 OP patients compared with 15 HC, and also in an OP mouse model. An increase of the expression levels of miR-338 cluster members was observed both in sera of OP patients compared with HC and ovariectomized mouse, and ROC analysis confirmed their diagnostic potential in determining patients with or without osteoporosis. Moreover, based on the results derived from a miR-338 cluster knockout mouse model, they observed that the leak of miR-338 promoted an increase of bone volume, thus reducing or preventing osteoporosis after ovariectomy. In this regard, they found that this cluster could inhibit osteoblastic differentiation by targeting the estrogen-dependent *Runx2* and *Sox4* genes. According to these findings, the serum miR-338 cluster could be a potential diagnostic biomarker and therapeutic target for OP patients.

Sun et al. [67] investigated the miRNA expression profile in the plasma of OP patients with or without vertebral fractures compared with HC. First, they profiled a miRNA array analysis comparing a panel of 384 miRNAs between 12 OP patients (six with and six without vertebral fractures) and six HC. Following qPCR validation on a greater independent population composed of 48 OP patients (24 with and 24 without vertebral fractures) and 24 HC, they identified that the plasma expression levels of miR-19b-3p were significantly lower in the OP group compared to HC. Furthermore, its AUC values showed the highest specificity and sensitivity values for differentiating between OP patients either with or without vertebral fractures and HC. In vitro, miR-19b-3p expression was significantly upregulated during osteogenic differentiation of hMSCs and MC3T3-E1 cells. In addition, they observed that this miRNA could play an important role in promoting such a process, possibly through the regulation of the PTEN/pAKT/Runx2 pathway. In vivo, miR-19b mimic treatment in ovariectomized mouse models prevented bone loss. Taken together, these data suggest the potential of miR-19b-3p as a clinical therapeutic target for treating osteoporosis.

The aim of a study by Zarecki et al. [68] was to analyze the expression levels of 21 selected miRNAs for their involvement in bone metabolism, in the serum of 39 subjects with low BMD and no vertebral fractures, 26 patients with vertebral fractures and low BMD without any treatment for osteoporosis, 19 patients with vertebral fractures and low BMD but receiving treatment for osteoporosis (i.e., alendronate, risedronate, calcium carbonate, and vitamin D3), and 42 HC, by using qPCR. Out of 21 tested miRNAs, seven miRNAs (miR-375, miR-532-3p, miR-19b-3p, miR-152-3p, miR-23a-3p, miR-335-5p, and miR-21-5p) were statistically higher in patients with vertebral fractures and low BMD compared with subjects with low BMD and no fractures or compared with HC, regardless of osteoporosis treatment. Moreover, they observed a total of 24 significant correlations between 11 miRNAs (miR-451a, miR-188-5p, miR-19b-3p, miR-486-3p, miR-550a-3p, miR-106b-5p, miR-144-3p, miR-29b-3p, miR-96-5p, miR-532-3p, and miR-30e-5p) and specific BTMs (PINP, osteocalcin, bone ALP, and CTX), suggesting their possible involvement in bone metabolism or fracture healing.

Wnt pathway is a critical regulator of bone formation, thus its disequilibrium could be involved in bone loss and consequent fracture events in OP patients. Bolamperti et al. [69] first analyzed the expression of genes involved in Wnt signaling, osteogenesis and adipogenesis processes in bone tissues derived from 25 women with femoral neck fracture and 29 nonfractured osteoarthritic patients (OA). The results showed a decrease in the expression of genes associated with osteogenesis in the fractured group compared to the OA group. Therefore, they investigated the expression levels of four miRNAs (miR-130a, miR-29a, miR-22, and miR-204) involved in the control of the osteogenic process in the serum samples of the same population, as well as in the bone specimens. Among these, the expression levels of miR-130a were significantly higher in the serum of patients with femoral fractures, while those of miR-204 displayed an opposite trend. Conversely, miR-29a and miR-22 were not differentially expressed between the two groups in both tissue and serum samples. In conclusion, the authors assumed that there is a response to the fracture event starting in serum, including a variation of miRNAs profile, which promotes an osteogenic effect.

Makitie et al. [70] studied the expression profile of 192 serum miRNAs in 12 patients carrying a mutation in exon 4 of *Wnt1* compared with 12 individuals mutation-negative assayed by qPCR. The expression levels of two miRNAs (miR-18a-3p, and miR-223-3p) were significantly higher, while six miRNAs (miR-22-3p, miR-31-5p, miR-34a-5p, miR-143-5p, miR-423-5p, and miR-423-3p) were lower in patients with *Wnt1* mutation compared with HC. Of these deregulated miRNAs, miR-22-3p, miR-34a-5p, and miR-31-5p have been reported able to inhibit the Wnt1 pathway. In conclusion, these identified miRNAs reflecting *Wnt1* mutation status could be potential biomarkers for the diagnosis and treatment of osteoporosis.

Two interesting studies assessed the reliability of a miRNA-specific bone panel developed by TamiRNA GmbH, the OsteomiR^TM^ test, which can be used for individual analysis of 19 serum miRNA biomarkers using qPCR, as a possible novel tool for screening and monitoring fracture risk. Walter et al. [71] evaluated the cost-effectiveness of the OsteomiR^TM^ test by comparing women who were either not subject to fracture risk assessment or monitoring, or who had been screened and monitored using either DXA alone, FRAX alone, or the OsteomiR^TM^ test, in a cohort of Austrian women without prior fractures, by using the Markov probabilistic model. Overall, the authors concluded that fracture risk assessment and monitoring based on miRNAs provides both a better fracture risk assessment and saves costs compared to the existing tools of fracture risk prediction, even if the OsteomiR^TM^ test is not intended as a replacement, but to complement the standard of care.

Likewise, Ladang et al. [72] studied the capacity of the OsteomiR score, based on measuring 10 out of the 19 miRNA biomarkers developed by TamiRNA GmbH (miR-335-5p, miR-152-3p, miR-127-3p, miR-320a, miR-144-5p, miR-582-5p, miR-17-5p, miR-375, miR-188-5p, and miR-141-3p), to estimate the fragility fracture risk in the serum of 17 individuals who had fractured within three years from the collection time versus 16 HC without a history of fractures during the same time span. They observed not only an OsteomiR score higher in osteopenic and OP patients compared with HC, but also that this tool permits prediction of fracture events with more sensitivity than FRAX, underlining the possible existence of a miRNA fracture signature several years before the fracture occurrence. Therefore, they assumed that implementation of the OsteomiR score alone or in combination with FRAX could aid the improvement of fracture risk assessment.

### 2.2. Diabetic Patients

Diabetes mellitus is a metabolic disorder caused by insufficient or no insulin production by the pancreas or by an impaired response to insulin, which can lead to bone loss, referred to clinically as secondary osteoporosis, resulting in an increased risk of bone fractures. Given that the current clinical practices underestimate the fracture risk in type 2 diabetes (T2D) patients, Heilmeier et al. [73] have attempted to identify a miRNA signature capable of discriminating fractured patients from nonfractured subjects in a human study of T2D and postmenopausal osteoporosis. They profiled a low-density qPCR array analysis to identify the most differentially expressed serum miRNAs able to distinguish the fractured patients from nonfractured controls in two distinct cohorts: the first consisted of T2D women with fractures (DMFx, *n* = 20) and without a history of fracture events (DM, *n* = 20); the second consisted of 20 nondiabetic OP patients with fractures and 20 HC. Forty-eight differentially expressed miRNAs were identified between DMFx and DM, while 23 miRNAs were differentially expressed between fractured OP patients and HC. Six miRNAs showed the same expression pattern between the T2DM and OP groups, and in particular, three miRNAs were upregulated (miR-550a-5p, miR-330-3p, and miR-203a) while three miRNAs were downregulated (miR-1908, miR-369-3p, and miR-382-3p). Based on the top 10 candidate 4-miRNA-models with the highest AUC values, the most frequently found miRNAs were miR-382-3p, miR-550a-5p, and miR-96-5p for the T2DM group and miR-188-3p, miR-382-3p, miR-942 for the OP group. Then, three miRNAs (miR-550a-5p, miR-188-3p, and miR-382-3p) were selected for further analysis, because they were the most abundant miRNAs found among the top 10 ranking miRNAs, and according to their fold changes and *p*-values. The authors performed in vitro analysis to investigate the potential role of the previously found miRNAs on osteogenesis, adipogenesis, and cell proliferation processes. miR-382-3p positively regulated osteogenic differentiation and acted as a negative regulator of adipogenesis, while miR-550a-5p was a potent inhibitor of osteogenesis and adipogenesis. Instead, no miRNAs exhibited effects on cell proliferation. In summary, they revealed miR-550a-5p and miR-382-3p as potential candidates that are indicative of the fragility status in the diabetic cohort, and miR-382-3p and miR-188-3p as the most discriminative for the osteoporosis-associated fractures.

In another study, Grieco et al. [74] evaluated, for the first time, the differentially expressed miRNAs in the serum samples of 15 type 1 diabetes (T1D) patients and 14 HC assayed by qPCR. Out of six miRNAs (miR-21, miR-24, miR-27a, miR-148a, miR-214, and miR-375), previously associated with TD1 and bone metabolism in the literature, they observed an increase in serum expression levels of miR-148a-3p and miR-21-5p in T1D patients compared with the controls. In addition, they made a correlation analysis between the identified miRNAs and the main parameters of bone metabolism, which resulted in a significant association between miR-148a and BMD values and PTH circulating levels, and miR-21-5p to Bone Mineral Content-Femur. Taken together, these findings suggest their role as potential biomarkers of bone fragility in T1D.

### 2.3. Antiosteoporosis Treatment

Denosumab (Dmab) and Teriparatide (TPDT) are currently approved as antiosteoporotic medications. Dmab is an agent that inhibits bone resorption by binding RANKL, while TPDT is a parathyroid hormone analog that stimulates bone formation. Given that a rapid loss of BMD with an increase of bone fractures following the end of treatment with Dmab was previously reported, Anastasilakis et al. [75] compared the clinical and biochemical parameters of five patients suffering from vertebral fractures after cessation of Dmab treatment (Dmab/Fx+), five fractured pharmacologically untreated patients (Fx+), and five women who had not sustained any vertebral fractures following discontinued Dmab treatment (Dmab/Fx−). The expression levels of miR-503 and miR-222-2 were significantly lower in the serum of Dmab/Fx+ and Dmab/Fx− patients compared with the Fx+ group. Both these miRNAs were associated with increased expression of genes involved in osteoclastogenesis and osteoclast activity, revealing a specific miRNA signature capable of discriminating patients suffering fractures associated with cessation of Dmab compared to fractured treatment-naive patients. However, it does not have any predictive power in identifying patients at highest risk for such fractures.

In another study, the same research group [76] investigated the effect of Dmab and TPDT on the serum expression levels of specific miRNAs that are known to be associated with bone metabolism. Consequently, Anastasilakis et al. analyzed the expression profile of miR-21-5p, miR-23a-3p, miR-24-2-5p, miR-26a-5p, miR-27a, miR-29c-3p, miR-33-3p, miR-124-3p, miR-133a-3p, miR-135b, miR-218-5p, miR-222-5p, miR-335-5p, miR-422a, miR-503, and miR-286 at 3 and 12 months in the serum of postmenopausal women with low bone mass who received either Dmab (*n* = 30) or TPDT (*n* = 30) by using qPCR. In the TPDT treated patients, miR-33-3p and miR-133a-3p levels were significantly decreased after 3 and 12 months, respectively. In addition, they found that the serum levels of miR-124-3p were inversely correlated with BMD values at 12 months, and relative serum expression of miR-24-3p and miR-27a was correlated with changes in BTMs. By contrast, in the Dmab treatment group, no significant change in miRNA serum levels was observed during treatment, while a correlation between six miRNAs (miR-21-5p, miR-23a-3p, miR-26a-5p, miR-27a, miR-222-5p, and miR-335-5p) and changes in BTMs was observed. Taken together, these findings show that significant changes in serum miRNA expression occur during TPDT and Dmab treatments.

Table 1 summarizes information about c-miRNAs as candidate biomarkers in bone fragility. 

## 3. miRNAs in Human Circulating Monocytes

Monocytes are the precursors of osteoclasts and represent a valid model for studying bone-related diseases. miRNAs in circulating monocytes, in common with c-miRNAs, may be used as potential noninvasive biomarkers in osteoporosis. In this regard, Wang et al. [77] isolated monocytes from 10 patients with high BMD and 10 patients with low BMD, and subsequently profiled a miRNA array analysis to identify differentially expressed miRNAs in circulating monocytes between these two groups. Among the 365 miRNAs, the expression levels of two miRNAs (miR-133a and miR-382) were significantly higher in the low BMD group compared to the high BMD group. Following the qPCR validation stage, only the miR-133a was significantly upregulated in the low BMD patients compared with the high BMD group. In conclusion, the authors proposed miR-133a in circulating monocytes as a candidate biomarker for osteoporosis.

The same authors [78] sought to identify other potential miRNA biomarkers in circulating monocytes for postmenopausal osteoporosis. They analyzed the expression levels of miRNAs by using the TaqMan miRNA array followed by qPCR validation in monocytes isolated from peripheral blood of 10 individuals with high BMD and 10 low BMD. Among the four differentially expressed miRNAs (miR-27b, miR-422a, miR-151, and miR-152) from the TaqMan array analysis, only miR-422a levels were significantly upregulated in the monocytes derived from the high BMD group compared with the low BMD group in the validation phase. Overall, Cao et al. suggested miR-422 as a potential cellular miRNA biomarker for postmenopausal women suffering from osteoporosis.

Jimenez-Ortega et al. [79] investigated the expression profile of miRNAs in monocytes isolated from the whole blood of six OP patients compared with six HC. First, they profiled a panel of 2.578 miRNAs by using microarray analysis to identify the differentially expressed miRNAs between patients and controls according to the fold change and *p*-value. Among 35 differentially expressed miRNAs, three miRNAs (miR-1270, miR-548x-3p, and miR-8084) were markedly upregulated in OP patients; these were subjected to validation, assayed by qPCR. Only the expression levels of miR-1270 were significantly higher in OP patients compared to HC. In addition, bioinformatic analysis revealed that this miRNA interacted with several bone-related target genes, in particular, *interferon regulatory factor 8* (*IRF8*), a gene associated with osteoclastogenesis, whose reduced expression in circulating monocytes could result in a low BMD, thus contributing to the development of osteoporosis. In conclusion, they suggested that miR-1270 had a potential viable as a biomarker for postmenopausal osteoporosis.

The same research team [80] identified four upregulated miRNAs (miR-708-5p, miR-34b-5p, miR-3161, and miR-328-5p) and two downregulated miRNAs (miR-4422 and miR-939-3p) in monocytes isolated from peripheral blood of seven OP patients, compared with seven HC, by using small RNA-sequencing. Following qPCR validation, only miR-708-5p was found significantly upregulated in OP women. By bioinformatic analysis, they showed that the target genes of this miRNA are related to the osteoclastogenesis process, such as *AKT1*, *AKT2*, *PARP1*, *FKBP5*, and *MAP2K3*, which were found downregulated in the OP group, in accordance with previous microarray analysis. This evidence indicated miR-708-5p as a potential biomarker for osteoporosis in Mexican postmenopausal women.

To identify biomarkers relative to osteoporosis disease, Zhang et al. [81] characterized the competing endogenous RNA (ceRNA) regulatory networks of mRNAs, miRNAs, and long noncoding RNAs (lncRNAs) from microarray data obtained from the Gene Expression Omnibus database. They took into account the gene expression profile from circulating monocytes of five subjects with high hip BMD compared with five subjects with low hip BMD. Bioinformatics and functional enrichment analysis showed that 297 mRNAs, 151 lncRNAs, and 38 miRNAs (miR-1281, miR-532-5p, miR-574-3p, miR-574-5p, miR-340-5p, miR-589-5p, miR-410-3p, miR-130b-5p, miR-376a-3p, miR-27b-5p, miR-411-3p, miR-1304-5p, miR-30a-3p, miR-34a-5p, miR-628-5p, miR-96-3p, miR-192-3p, miR-564, miR-767-5p, miR-202-5p, miR-1286, miR-1470, miR-29b-3p, miR-3130-5p, miR-624-5p, miR-340-3p, miR-490-5p, miR-369-5p, miR-1273c, miR-376b-3p, miR-194-5p, miR-874-3p, miR-1260a, miR-3128, miR-3177-3p, miR-378b, miR-3124-5p, and miR-92a-1-5p) were differentially expressed between these two groups. These results revealed a complex network of mRNA and noncoding RNA molecules involved in the pathogenesis of osteoporosis, which could be candidate biomarkers or targets in the diagnosis and management of osteoporosis.

Table 2 summarizes information about differentially expressed miRNAs in circulating monocytes isolated from OP patients.

## 4. Discussion

An ideal biomarker is defined by the World Health Organization (WHO) as “any substance, its products, structure or process that can be measured in the body and that influences or predicts the incidence of outcome or disease” [82]. They are essential for the diagnosis, prognosis, and development of treatment strategies in virtually all diseases, including osteoporosis, one of the major causes of bone fracture. Osteoporosis is a systemic bone disease that predominantly affects postmenopausal women, leading to a reduction in bone mass, with consequent increased risk of bone fragility and susceptibility to fracture [83]. It has been estimated that there are approximately nine million osteoporotic fractures annually worldwide [84]. As a result, osteoporosis has become an important health problem, whose consequences are associated with an increase in morbidity and mortality rates, and financial burden.

Nowadays, there are several diagnostic tools used in clinical practice for fracture risk assessment, such as DEXA, WHO-FRAX score, QCT, and BTMs. However, there are some limitations that hamper their utility in the clinical setting [17]. Therefore, there is a great need to find novel easily measurable biomarkers that can assist the clinician in making decisions in this field [17,85].

Due to the multifactorial nature of osteoporosis, it has been assumed that genetic factors alone are not able to clarify its development, implying that epigenetic mechanisms could be responsible for the loss of bone mass and susceptibility to bone fractures.

Epigenetics is a particular phenotypic inheritance mechanism that does not involve changes in DNA sequences. This mechanism includes processes such as DNA methylation, histone modifications, and gene silencing by noncoding RNA (ncRNA) activity [86].

The latter mechanism has attracted attention in the field of biomarker discovery during recent years, and in particular miRNAs. In fact, several studies have reported the crucial role of these small molecules in osteoblastogenesis and osteoclastogenesis processes, and that dysregulation in their expression profiles could markedly disrupt the balance of the bone homeostasis, highlighting their appeal as ideal biomarkers for osteoporosis and bone fracture risk [87]. The initial attempt to identify differentially expressed miRNAs in patients with discordant BMD status focused on the measurement of these molecules in circulating monocytes, osteoclast precursor cells demonstrated to be critical in the pathogenesis of osteoporosis, that could constitute a reliable model for the study of the etiology of osteoporosis [77,88]. As described in this review, few studies (Table 2) have currently evaluated the miRNA expression in circulating monocytes from OP patients and, in our opinion, this research should be considered in the future.

The scientific community is currently focusing its efforts on identifying specific and highly stable c-mRNAs. In fact, several c-miRNAs have been suggested as potential noninvasive diagnostic biomarkers since 2008, when Lawrie et al. described, for the first time, the upregulation of miR-21 in serum samples from patients affected by diffuse large B-cell lymphoma (DLBCL) [34]. Subsequently, it has been shown that mature miRNAs may be released in at least 12 different biofluids, paving the way for new possibilities in medicine [39]. These highly stable and quickly detectable circulating molecules could be helpful to establish a diagnosis prior to the appearance of clinical signs. Moreover, their expression profiles in biological fluids are also strongly dependent on physiological and pathological stimuli, reflecting the functional status of cells, and also giving information on disease progression [89]. In recent years, an increasing number of studies have revealed them as candidate biomarkers in several disorders, including different types of cancers and cardiovascular, neurological, gastrointestinal, and bone diseases, including osteoporosis [90,91]. Based on the current review, several c-miRNAs have been correlated to osteoporosis and the occurrence of osteoporotic fractures.

Recently, some evidence has also proven that anti-miRNA oligonucleotides (AMOs) could be used to suppress miRNA function, becoming suitable candidates for drug design, especially in cases where increased expression of miRNAs is correlated to the onset of a specific disease [92].

**Figure 2:**
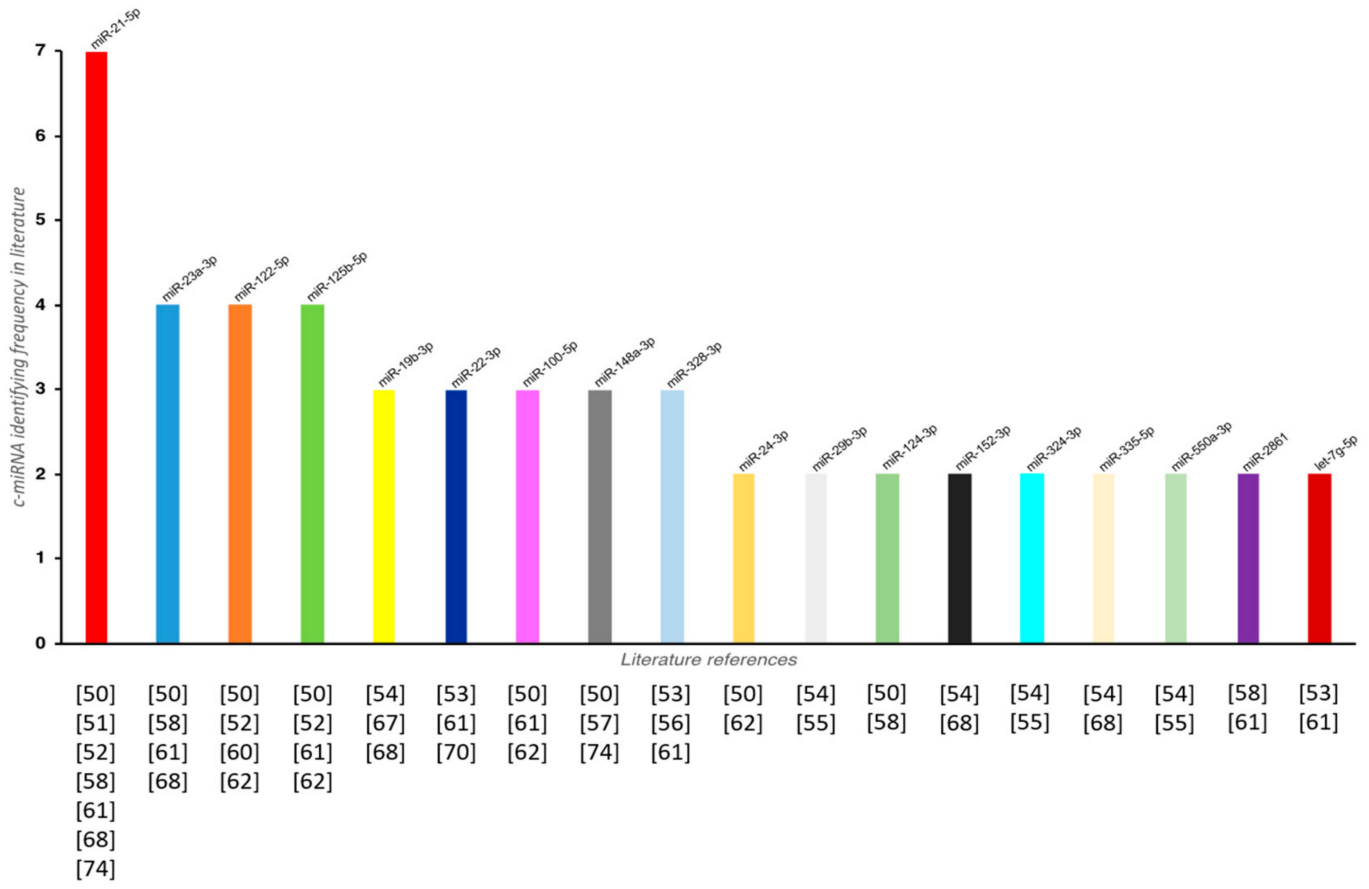
c-miRNAs literature distribution. The figure shows the most important c-miRNAs that have been suggested as potential novel biomarkers for bone fragility. These molecules are seeded based on their identifying frequency in the literature to date. Those with frequency = 1 are not shown.

The discovery of a panel of miRNAs in biological fluids could be helpful both for fracture risk assessment and for monitoring these patients. In fact, the presence of a c-miRNA signature years before altered bone status and before the manifestation of a fracture could be useful to better predict fracture risk compared to current tools. This would permit not only the possibility to address future patients towards efficient antiosteoporotic treatments, but also to reduce in a meaningful way fracture risk and the related costs for patient management. Several studies have reported an altered expression level of c-miRNAs in patients affected by osteoporosis and in those who presented an osteoporosis-related fracture (Table 1). Figure 2 shows the c-miRNAs that have resulted to be the most commonly linked to a decrease of BMD and impaired bone strength in these patients.

The clinical utility of the detection of c-miRNAs in assessing bone fragility has not yet been determined. These molecules have benefits derived from their application potential in the clinical setting as a result of their remarkable stability once collected, and easy noninvasive availability compared to bone biopsies [17]. Further studies should be conducted to define their place in medical programs as well as to discover how the c-miRNA profile could impact medical decisions.

Different aspects should be taken into account for the identification of steady, noninvasive, and disease-specific biomarkers. In fact, the scientific community is aware that c-miRNA expression levels are affected by the interindividual differences related both to the lifestyle and the health status of the patient. It is also known that each platform used for evaluating c-miRNAs, such as microarrays, NGS, qPCR, impacts their quantification. The first method allows for exploration of the expression profiling of a specific set of known c-miRNAs. Nevertheless, this technology cannot be used for their absolute quantification and requires a great amount of total RNA starting material [93]. The NGS platform is the only tool that also allows the identification of novel miRNAs. However, bioinformatics support is required for the data analysis and, as for the microarray technology, this detection system requires large amounts of input RNA [93]. To date, qPCR assay is the gold standard platform for profiling the relative expression of c-miRNAs due to its high accuracy, lower required template, and its more reliable results. In fact, the results obtained from both microarray and NGS analysis require an additional validation step in qPCR [94]. However, one of the major challenges for c-miRNA expression analysis by using this method regards the best RG for normalizing c-miRNA levels [94]. Indeed, no consensus exists so far regarding the right single or panel of RGs for normalizing qPCR data. This latter is essential to interpret and compare studies and outcomes reported by different laboratories. In that sense, further studies are necessary to select the most stable endogenous circulating molecules to normalize miRNA expression levels into biological fluids.

In view of this, the scientific community is still far from the identification of a specific c-miRNA signature in patients with bone fragility. This signature could lead to the optimization of risk assessment as well as treatment of bone fragility, either alone or in combination with other clinical parameters. As described by Hackl et al. [17], one possible way to identify a panel of c-miRNAs as biomarkers for diagnosis, prognosis, patient stratification, and personalized medicine, could be to develop a systematic biomarker screening protocol, which does not exclude miRNAs a priori (i.e., full miRNome NGS analysis). Candidate miRNAs should be validated in prospective cohort studies of fractured patients within 5-10 years from their recruitment, in order to determine both their reference and threshold values for the clinical management of the disease.

This review provides an overview of the candidate c-miRNAs that could be novel biomarkers, in addition to the pre-existing ones, to make not only more accurate and earlier diagnoses years prior to bone fracture, but also for the future development of RNA antagomirs-based strategies, in order to treat fragility fractures and bone loss in these patients. To reach this objective, these molecules will have to be validated and confirmed in independent studies before reaching application in clinical settings.

## Figures and Tables

**Table 1:** Overview of c-miRNAs as candidate biomarkers in bone fragility.

Biological Fluids	Study Population	Platforms for miRNAs Expression Profiling	Identified Candidate c-miRNAs	ROC Curve Analysis Data (AUC Value)	RGs Used for Normalizing qPCR Data	Study
Serum	OP (40), HC (40)	Prescreening: miRNA PCR arrays Validation: qPCR	miR-21-5p (↑), miR-23a-3p (↑), miR-24-3p (↑), miR-93 (↑), miR-100-5p (↑), miR-122-5p (↑), miR-124-3p (↑), miR-125b-5p (↑), miR-148a-3p (↑)	OP vs. HC 0.63 (miR-21-5p), 0.63 (miR-23a-3p), 0.63 (miR-24-3p), 0.68 (miR-93), 0.69 (miR-100-5p), 0.77 (miR-122-5p), 0.69 (miR-124-3p), 0.76 (miR-125b-5p), 0.61 (miR-148a-3p)	Average of SNORD96A and RNU6	[50]
Plasma	Osteopenic (40), OP (40), HC (40)	qPCR	miR-21-5p (↓), miR-133a (↑)	/	miR-16	[51]
Serum	OP with bone fractures (23), HC (17)	Prescreening: PCR Panel Validation: qPCR	miR-122-5p (↑), miR-125b-5p (↑), miR-21-5p (↑)	OP fracture vs. HC 0.87 (miR-122-5p), 0.76 (miR-125b-5p), 0.87 (miR-21-5p)	miR-93-5p	[52]
Serum	OP with fractures (19), HC (18)	Prescreening: qPCR Validation: qPCR	miR-22-3p (↑), miR-328-3p (↓), let-7g-5p (↓)	/	Global mean Ct value	[53]
Serum	Low-traumatic fractures (36), HC (39)	qPCR	miR-152-3p (↓), miR-30e-5p (↓), miR-140-5p (↓), miR-324-3p (↓), miR-19b-3p (↓), miR-335-5p (↑), miR-19a-3p (↓), miR-550a-3p (↓), miR-29b-3p (↓)	OP fracture vs. HC 0.962 (miR-152-3p), 0.959 (miR-30e-5p), 0.947 (miR-140-5p), 0.95 (miR-324-3p), 0.944 (miR-19b-3p), 0.939 (miR-335-5p), 0.929 (miR-19a-3p), 0.909 (miR-550a-3p), 0.838 (miR-29b-3p)	Global mean Ct value	[54]
Serum	Low-traumatic fractures (36)	qPCR	miR-29b-3p (↓), miR-324-3p (↓), miR-550a-3p (↓)	/	Global mean Ct value	[55]
Serum	OVX rats, rhesus monkeys, women with normal BMD (19), osteopenia (7), OP (10)	Prescreening: miRNA PCR arrays Validation: qPCR	miR-30b-5p (↓), miR-103-3p (↓), miR-142-3p (↓), miR-328-3p (↓)	OP vs. HC 0.926 (miR-30b-5p), 0.796 (miR-103-3p), 0.95 (miR-142-3p)	miR-25-3p	[56]
Plasma	OP (17), HC (57)	qPCR	miR-148a-3p (↑)	/	Mean of miR-16-5p and let-7a-5p	[57]
Serum	OP with vertebral fractures (35) OP (35), HC (30)	qPCR	miR-124-3p (↑), miR-2861 (↑), miR-21-5p (↓), miR-23a-3p (↓), miR-29a-3p (↓)	OP vs. osteopenic 0.66 (miR-21-5p), 0.63 (miR-23a-3p), 0.61 (miR-29a-3p)	SNORD95A, SNORD96A, RNU6-2	[58]
Serum	Osteopenic (28), OP (46), OP with hip fractures (21), HC (42)	Prescreening: qPCR arrays Validation: qPCR	miR-23b-3p (↑), miR-140-3p (↑)	OP vs. HC: 0.69 (miR-23b-3p), 0.96 (miR-140-3p); OP fracture vs. HC: 0.8869 (miR-23b-3p), 0.92 (miR-140-3p)	RNU6	[59]
Serum and Plasma	Osteopenic with fractures (15), Osteopenic w/o fractures (61), OP with fracture (18), OP w/o fractures (33), HC (12)	Prescreening: qPCR arrays Validation: qPCR	miR-122-5p (↓), miR-4516 (↓)	OP vs. HC 0.727 (miR-4516) 0.757 (miR-4516 + miR-122-5p)	SNORD96A, RNU6-6P	[60]
Serum	OP (9), HC (9)	qPCR	let-7g-5p (↓), miR-133a-5p (↓), miR-328-3p (↓), miR-22-3p (↓), miR-2861 (↓), miR-518d-5p (↓), miR-10b-5p (↑), miR-21-5p (↑), miR-125b-5p (↑), miR-23-3p (↑), miR-100-5p (↑)	OP vs. HC 0.8944 (let-7g-5p), 0.8656 (miR-328-3p), 0.8728 (miR-10b-5p), 0.8875 (miR-100)	U6	[61]
Serum	OP (45), fractured non-OP (15)	qPCR	miR-24-3p (↑), miR-27a-3p (↑), miR-100-5p (↑), miR-125b-5p (↑), miR-122-5p (↑), miR-145 (↑), miR-144-3p (↓)	/	U6	[62]
Serum	OP w/o fractures (559), OP with fractures (123)	qPCR	/	/	UniSP6, Mean of each miRNA	[63]
Serum	OP fractures (217), HC (217)	qPCR	/	/	miR-191-5p, miR-222-3p miR-361-5p	[64]
Serum	NN (13), OO (46), SOP (15), SP (1)	qPCR	/	/	Geometric mean of miR-16-5p, miR-93-5p, miR-191-5p	[65]
Serum	OP (15), HC (15)	qPCR	miR-338-3p (↑), miR-3065-5p (↑)	OP vs. HC 0.74 (miR-338-3p), 0.87 (miR-3065-5p)	Cel-miR-39-3p	[66]
Plasma	OP with vertebral compression fractures (30), OP w/o vertebral fractures (30), HC (30)	Prescreening: miRNA array analysis Validation: qPCR	miR-19b-3p (↓)	OP w/o fracture vs. HC 0.9280 (miR-19b-3p) OP fracture vs. HC 0.9505 (miR-19b-3p)	U6	[67]
Serum	No vertebral fractures/Low BMD (35), Vertebral fractures/Low BMD receiving treatment for osteoporosis (17), Vertebral fractures/Low BMD w/o any treatment for osteoporosis (24), HC (40)	qPCR	miR-375 (↑), miR-532-3p (↑), miR-19b-3p (↑), miR-152-3p (↑), miR-23a-3p (↑), miR-335-5p (↑), miR-21-5p (↑)	/	UniSp4	[68]
Serum	Fragility femoral fractures (25), Osteoarthritis (25)	qPCR	miR-130a (↑), mi-204 (↓)	/	snU6	[69]
Serum	*WNT1* Mutation-positive OP (12), *WNT1* Mutation-negative OP (12)	qPCR	miR-18a-3p (↑), miR-223-3p (↑), miR-22-3p (↓), miR-31-5p (↓), miR-34a-5p (↓), miR-143-5p (↓), miR-423-5p (↓), miR-423-3p (↓)	/	Global mean Ct value	[70]
Serum	/	Markov model	OsteomiR^TM^ test	/	/	[71]
Serum	Fracture group (17), HC (16)	qPCR	OsteomiR° test	OP fracture vs. HC 0.687 (OsteomiR test)	UniSp4	[72]
Serum	DM (20), DMFx (20), nondiabetic OP women with fractures (20), HC (20)	Low-density qPCR array platform	miR-550a-5p (↑), miR-188-3p (↓), miR-382-3p (↓)	DMFX vs. DM: 10 candidate miRNAs panels (model of 4 miRNAs) have an AUC value between 0.922 and 0.965; OP vs. HC 10 candidate miRNAs panels (model of 4 miRNAs) have an AUC value between 0.972 and 0.991	Ct values were computed using the second derivative maximum method provided with the LC480 II software.	[73]
Serum	T1D (15), HC (14)	qPCR	miR-148a-3p (↑), miR-21-5p (↑)	/	miR-191, ath-miR-159a	[74]
Serum	Dmab/Fx+ (5), Fx + (5), Dmab/Fx− (5)	qPCR	miR-503 (↓), miR-222-2 (↓)	/	SNORD95A, SNORD96A, RNU6-2	[75]
Serum	Low bone mass women treated with either Dmab (30) or TPDT (30)	qPCR	miR-33-3p (↓) miR-133a-3p (↓)	/	SNORD95A, SNORD96A, RNU6-2	[76]

* Enrolled population is shown in parentheses.

**Table 2:** Overview of differentially expressed miRNAs in circulating monocytes isolated from osteoporotic (OP) patients.

Study Population	Platforms for miRNAs Expression Profiling	Identified Candidate miRNAs in Human Circulating Monocytes	ROC Curve Analysis Data (AUC Value)	Study
High BMD (10), low BMD (10)	Prescreening: miRNA array Validation: qPCR	miR-133a (↑)	/	[77]
High BMD (10), low BMD (10)	Prescreening: miRNA array Validation: qPCR	miR-422a (↑)	/	[78]
OP (6), HC (6)	Prescreening: microarray Validation: qPCR	miR-1270 (↑)	/	[79]
OP (7), HC (7)	Prescreening: small RNA-sequencing Validation: qPCR	miRN-708-5p (↑)	/	[80]
High hip BMD (5), low hip BMD (5)	Bioinformatic analysis from microarray data	38 miRNAs (see text)	/	[81]

* Enrolled population is shown in parentheses.
